# Intercropping with wheat lowers nutrient uptake and biomass accumulation of maize, but increases photosynthetic rate of the ear leaf

**DOI:** 10.1093/aobpla/ply010

**Published:** 2018-02-08

**Authors:** Fang Gou, Martin K van Ittersum, Antoine Couëdel, Yue Zhang, Yajun Wang, Peter E L van der Putten, Lizhen Zhang, Wopke van der Werf

**Affiliations:** 1 Centre for Crop Systems Analysis, Wageningen University, AK Wageningen, The Netherlands; 2 Plant Production Systems, Wageningen University, AK Wageningen, The Netherlands; 3 AGIR, Université de Toulouse, INRA, INPT, INP – EI PURPAN, chemin de Borde-Rouge, Castanet-Tolosan, France; 4 Agricultural Meteorology Department, College of Resources and Environmental Sciences, China Agricultural University, Beijing, China; 5 Chinese Academy of Sciences, Northwest Institute of Eco-Environment and Resources, Lanzhou, China

**Keywords:** Acclimation, nitrogen, phosphorus, plasticity, SPAD, stomatal conductance, *Triticum aestivum*, *Zea mays*

## Abstract

Intercropping is an ancient agricultural practice that provides a possible pathway for sustainable increases in crop yields. Here, we determine how competition with wheat affects nutrient uptake (nitrogen and phosphorus) and leaf traits, such as photosynthetic rate, in maize. In a field experiment, maize was planted as a sole crop, in three different intercrop configurations with wheat (a replacement intercrop and two add-row intercrops), and as a skip-row system with one out of each three maize rows omitted. Nitrogen and phosphorus uptake were determined at flowering and maturity. Specific leaf area, leaf nitrogen concentration, chlorophyll content and photosynthetic rate of the ear leaf were determined at flowering. Nitrogen and phosphorus concentrations were significantly lower in intercropped maize than in sole maize and skip-row maize at flowering, but these differences were smaller at maturity. At flowering, specific leaf area was significantly greater in intercrops than in skip-row maize. Leaf nitrogen concentration was significantly lower in add-row intercrops than in sole maize, skip-row maize or maize in the replacement intercrop. Leaf chlorophyll content was highest in sole and skip-row maize, intermediate in maize in the replacement intercrop and lowest in maize grown in add-row intercrops. On the contrary, photosynthetic rate was significantly higher in the replacement intercrop than in sole maize, skip-row maize and the intercrop with an additional maize row. The findings indicate that competition with intercropped wheat severely constrained nutrient uptake in maize, while photosynthetic rate of the ear leaf was not negatively affected. Possible mechanisms for higher photosynthesis rate at lower leaf nitrogen content in intercropped maize are discussed.

## Teaser

Maize and wheat are globally important food crops. The two species can be grown as an intercrop, with substantial land sparing in the order of 20 %, as expressed by a land equivalent ratio of ~1.2. Here, we study nutrient uptake and the photosynthesis rate of intercropped maize and show that nutrient uptake is constrained by competition with wheat, while the photosynthesis rate is not decreased, but—surprisingly—increased. Ecophysiological mechanisms potentially underlying the unexpected high photosynthesis rate in intercropped maize are discussed.

## Introduction

Intercropping is the cultivation of two or more crop species simultaneously in the same field (Vandermeer 1989). Due to complementary resource use in time and space among different species, intercropped plants usually achieve—on average across species—a greater yield per plant than plants of the same species in sole crops. As a result, the land equivalent ratio (LER, which is calculated as the sum of the relative yields of component species in an intercrop as compared to the yields of the sole crops) is greater than one (Lithourgidis *et al.* 2007; Malézieux *et al.* 2009; Li *et al.* 2013; Yu *et al.* 2015). When intercropped species have similar growing periods, the intercropping advantage is usually based on species differences in resource acquisition strategy or a decrease of crop failure probability, especially under low input conditions. The yield advantage decreases with an increase in inputs when the growing period of the intercropped species is the same (Yu *et al.* 2015). When intercropped species have different growing periods, e.g. in relay intercropping, the intercrop advantage is in part due to complementary resource use in time. Relay intercrops attain high LER especially at high input levels (Yu *et al.* 2015).

Wheat–maize relay intercropping is an example of a high input relay intercrop system that is practised by smallholder farmers in northwest China (Li *et al.* 2001b; Knörzer *et al.* 2009). In wheat–maize relay intercropping, spring wheat is sown as six rows-wide strips in March, while two rows of maize are sown in the bare strips between the wheat strips in April. Wheat is subsequently harvested in July and maize is harvested in September (Li *et al.* 2001b). Land equivalent ratio of this wheat–maize relay intercropping system varies from 1.1 to 1.6 (Li *et al.* 2001b; Song *et al.* 2006; Yang *et al.* 2011; Mu *et al.* 2013). However, when this system was tested under Dutch growing conditions, the LER was smaller than in northwest China, varying from 0.98 to 1.3 in 2 years (Gou *et al.* 2016).

While land productivity of intercropping differs under Chinese and Dutch growing conditions, the growth patterns of intercropped wheat and maize are similar in these two countries. Under both growing conditions, intercropped wheat had a yield advantage compared to sole wheat, due to much better growth in wheat border rows in the intercrop. Border row wheat had more tillers per plant and greater kernel number per ear than wheat in sole crop or wheat in the inner rows of wheat strips in the intercrop (Li *et al.* 2001b; Gou *et al.* 2016; Zhu *et al.* 2016). Maize is the later sown crop in wheat–maize relay intercropping, both in China and the Netherlands. As a result of competition with wheat, early growth of maize is slower in the intercrop than in a sole crop, both in China (Li *et al.* 2001b) and in the Netherlands (Gou *et al.* 2016). Zhu *et al.* (2014) found that intercropped maize in the Netherlands had a lower leaf appearance rate than sole maize. The comparative biomass and yield of intercropped versus sole maize differed however between the two locations. Li *et al.* (2001a) found that intercropped maize attained higher yield per plant than sole maize in northwest China but Gou *et al.* (2016) found that under Dutch growing conditions intercropped maize had a lower biomass per plant at maturity than sole maize.

In China, intercropped maize plants show recovery growth after wheat harvest, and on average, over the whole season, intercropped maize plants experience less competition for resources than maize plants in a sole crop. The severe competition from wheat during early maize growth is thus more than compensated after wheat harvest, when intercropped maize is growing at a lower density than sole maize, resulting in a relaxation of competition and greater access to light, water and nutrient resources per plant in the intercrop as compared to the sole crop. This pattern of strong competition during early growth, changing into relaxed competition and recovery during later growth was named the ‘competition-recovery production principle’ by Zhang and Li (2003). The strength of the ‘recovery growth of maize after wheat harvest’ was found to be weaker under Dutch growing conditions (Gou *et al.* 2016). This weaker recovery may be due to less suitable climatic conditions for maize growth in the Netherlands (lower temperature and radiation levels than in Gansu) but it could also be related to lower nutrient application levels in the Netherlands as compared to China (Gou *et al.* 2016). In the Netherlands, under the influence of environmental protection laws and obligatory nutrient bookkeeping at farm level, farmers provide crops with tailored fertilizer amounts, whereas farmers in China tend to over-fertilize to achieve maximum yields (Qiao *et al.* 2006; Huang *et al.* 2008). Intercropped maize was found to have a lower radiation use efficiency (RUE) over the whole season than sole maize (Gou *et al.* 2017).

In this paper, we aim to find out whether there are differences in nutrient (nitrogen and phosphorus) uptake and leaf traits between sole maize and intercropped maize that may explain the comparatively shallow recovery growth and low RUE of intercropped maize under Dutch growing conditions. We hypothesized that (i) intercropped maize would have lower nutrient uptake per plant and lower nutrient concentration in tissues than sole maize. Such lower nutrient concentration might explain the lower yield per plant and smaller RUE in intercropped maize. We also hypothesized that (ii) intercropped maize has a lower specific leaf nitrogen (SLN) than sole maize, due to competition for nutrients with wheat. Third, we hypothesized that (iii) intercropped maize has a higher specific leaf area (SLA) (‘thinner’ leaves) than sole maize due to shade avoidance during the early growth of maize, when the plants are shaded by earlier sown wheat. Finally, we hypothesized that (iv) lower nitrogen content, SLN and SLA of maize leaves in an intercrop would result in a lower rate of leaf photosynthesis. The overall aim of the paper is to propose an explanation for the lower yield and RUE of intercropped versus sole crop maize under Dutch growing conditions.

## Methods

### Experimental design

**Figure 1.**
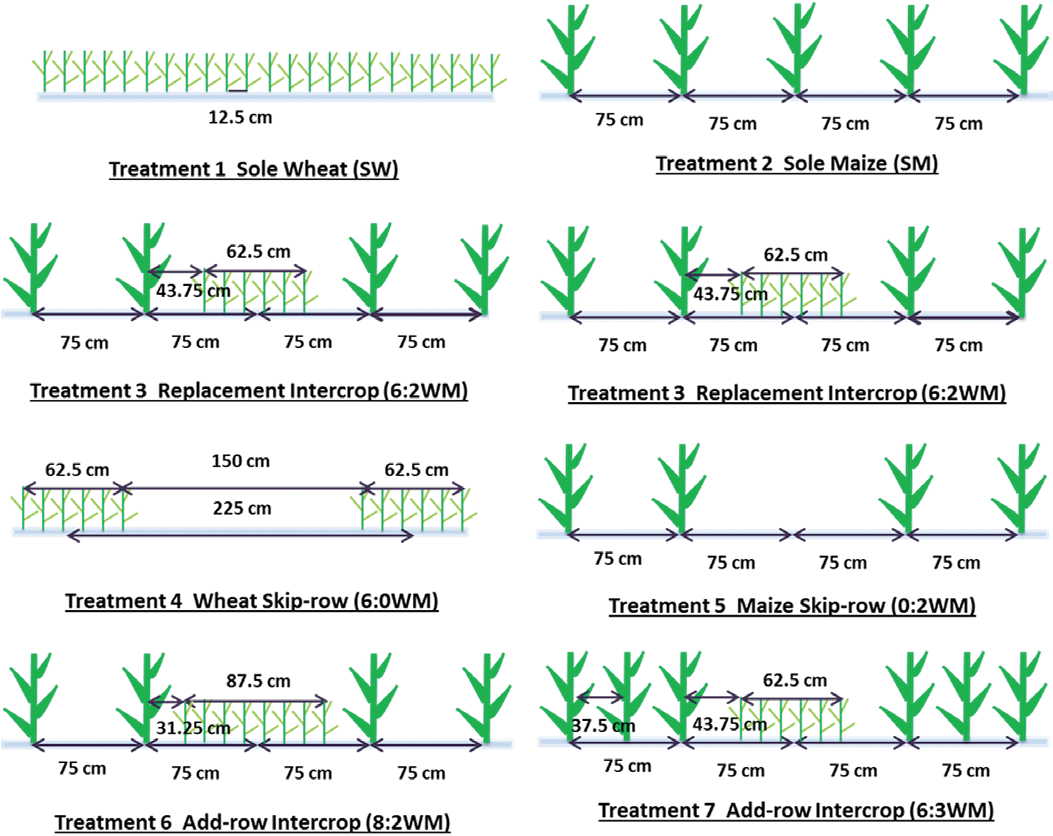
Schematic illustration of row placement of wheat and maize in different experimental planting patterns (reproduced with permission from Gou *et al.* [2016]).

Measurements were made in a field experiment in Wageningen, the Netherlands (51°59′20″N, 5°39′16″E) in 2014. The experiment had seven crop configurations as treatments: two sole crops, three intercrops and two skip-row treatments: sole wheat (SW) and sole maize (SM), replacement intercrop (6:2WM), skip-row wheat and maize (6:0WM, 0:2WM), and add-row intercrops (8:2WM, 6:3WM) (Table 1; Fig. 1). Details of the experimental design and crop management are given in Gou *et al.* (2016).

**Table 1.** Crop configuration parameters in seven treatments. Overall densities are densities per unit width or area of the whole intercrop. *The distance between the adjacent wheat and maize rows. The table is modified with permission from Gou *et al.* (2016).

Treatment	Row distance (cm)			Number of rows per 225 cm		Overall row density (rows per m)		Overall planting density (plants per m^2^)		Relative density		
	Wheat	Maize	Distance*	Wheat	Maize	Wheat	Maize	Wheat	Maize	Wheat	Maize	Total
SW	12.5	–	–	18	–	2.67	–	250	–	1	–	1
SM	–	75	–	–	3	–	1.33	–	10	–	1	1
6:2WM	12.5	75	43.75	6	2	2.67	0.89	83.3	6.7	0.33	0.67	1
6:0WM	12.5	–	–	6	–	2.67	–	83.3	–	0.33	–	0.33
0:2WM	–	75	–	–	2	–	0.89	–	6.7	–	0.67	0.67
8:2WM	12.5	75	31.25	8	2	3.56	0.89	111.1	6.7	0.44	0.67	1.11
6:3WM	12.5	37.5	43.75	6	3	2.67	1.33	83.3	10	0.33	1	1.33

Soil at the experimental site was sandy with 3.1 % organic matter and a C/N ratio in the organic matter of 14. Spring wheat (variety ‘Tybalt’) was sown on 13 March and harvested on 4 August, while maize (variety ‘Atrium’) was sown on 7 May and harvested on 23 September. A randomized complete block design with four replicates was used. Plots were 7.5 m wide by 23 m long. The row orientation was approximately north-south. Crop management in the experiments aimed at meeting crop demand for water and nutrients, and control of yield-reducing factors through adequate weed, pest and disease management. Fertilizer was applied homogeneously throughout the experiment. Before wheat sowing, K_2_SO_4_·MgSO_4_ and Ca(H_2_PO_4_)_2_·H_2_O were applied to supply phosphorus, potassium and calcium. Total available nitrogen was 200 kg N ha^−1^, including 7 kg N of soil mineral nitrogen at sowing of wheat, an estimated 25 kg N from winter cover crop decomposition (white mustard, *Sinapis alba*) and 168 kg N from nitrogen fertilizer (NH_4_NO_3_·CaMg(CO_3_)_2_). Weeds were controlled mechanically before wheat emergence and chemically thereafter. Supplementary water was applied during the growing season according to the estimation of weekly plant water demand (evapotranspiration) and precipitation.

### Determination of nutrient uptake

We determined the above-ground biomass of maize at flowering and maturity, and the grain yield at maturity (Gou *et al.* 2016, 2017). The dry matter of each organ (leaf, stem, ear, grain and straw) was ground and the nitrogen and phosphorus were measured. Samples were digested with a mixture of H_2_SO_4_–Se and salicylic acid (Novozamsky *et al.* 1983). The actual digestion was started by H_2_O_2_ and in this step most of the organic matter was oxidized. After decomposition of the excess H_2_O_2_ and evaporation of water, the digestion was completed by concentrated H_2_SO_4_ at elevated temperature (330 °C) under the influence of Se as a catalyst. In these digests total N and P were measured spectrophotometrically with a segmented-flow system (Auto-analyzer II, Technicon). The analyses were done at the department of Environmental Sciences, subdivision Nature Conservation and Plant Ecology, Wageningen University and Research, the Netherlands.

### Photosynthesis measurements

At flowering, we measured photosynthesis of the ear leaf (usually the ninth leaf) from 0900 to 1700 h using a Li-6400XT portable photosynthesis system (Licor Inc., Lincoln, NE, USA). The measurements were conducted during 3 weeks in each of four blocks of the field experiment. In Weeks 29 and 30 (week of the year), four plants per row were measured in each treatment plot per block, using an adaptation time of 5 min; and in Week 31, two plants per row in each treatment plot per block were measured using an adaptation time of 15 min **[see Supporting Information—Table S1]**. In the treatments SM, 0:2WM, 6:2WM and 8:2WM, two rows of maize plants were measured in each plot; in the 6:3WM treatment, plants in each of three maize rows were measured in each plot. Measurements were conducted at a constant light level of 1000 μmol m^−2^ s^−1^ and a constant CO_2_ level of 400 μmol mol^−1^. Leaf temperature during measurements was ~27 °C. Besides photosynthetic rate, stomatal conductance was also investigated which reflects water availability during the photosynthetic process.

### Leaf trait measurements

After the photosynthesis measurements, the leaves were cut and brought to the laboratory for further analyses. A section of ~20 cm length was cut from each maize leaf, and the midrib was removed. The greenness of the leaf, as a proxy of chlorophyll content, was measured using a SPAD 502 Plus Chlorophyll Meter (SPAD-502, Minolta Camera, Tokyo, Japan). Twelve different points on each blade were measured. The leaf area was measured using a leaf area meter (LI-3100 Area Meter, USA). Afterwards, the leaves were dried in an oven at 70 °C for 24 h to determine dry matter content. Finally, the nitrogen concentration of each oven-dried leaf was determined using the Kjeldahl method (Novozamsky *et al.* 1983). The oven-dried leaves were ground, and 0.1 g fine sample was digested in a mixture of concentrated H_2_SO_4_ and H_2_O_2_, and then the digests were analysed by a Kjeldahl device (KDY 9820, Tongrunyuang, China).

### Data analysis

Nitrogen and phosphorus uptake, and nitrogen and phosphorus concentration were analysed separately at flowering and at maturity. Treatment and block were factors in this analysis. The leaf traits and photosynthetic rate were analysed separately for each week of measurements, while treatment and block were factors in the analysis for each week. Leaf traits and photosynthetic rate were also analysed combining data for all the 3 weeks, with treatment, week and block as factors in the analysis. In the above analyses, pairwise comparisons were made between treatments. ANOVA (*P* = 0.05) and Tukey’s HSD were done using the ‘stats’ package of the R programming language (R Core Team 2015).

Partial LER for maize biomass and yield, partial nitrogen uptake equivalent ratio (*p*NER) and partial phosphorus uptake equivalent ratio (*p*PER) for maize were calculated using the following equations:

$$pLER=\frac{Y_{i}}{Y_{s}}$$ (1)

$$pNER=\frac{N_{u,i}}{N_{u,s}}$$ (2)

$$pPER=\frac{P_{u,i}}{P_{u,s}}$$ (3)

where *p*LER is partial LER for biomass or yield, *Y*_i_ (t ha^−1^) is biomass or yield in intercrop for species *i*, *Y*_s_ (t ha^−1^) is biomass or yield in sole crop for the same species; *p*NER is partial nitrogen uptake equivalent ratio, N_u,i_ (kg N ha^−1^) is nitrogen uptake in the intercrop for species *i*, while N_u,s_ (kg N ha^−1^) is nitrogen uptake in the sole crop for the same species; *p*PER is partial phosphorus uptake equivalent ratio, P_u,i_ (kg P ha^−1^) is phosphorus uptake in the intercrop for species *i*, while P_u,s_ (kg P ha^−1^) is phosphorus uptake in the sole crop for the same species.

To diagnose maize nitrogen status, we used an allometric relationship between critical nitrogen concentration (%N_c_) and shoot biomass (*W*, t ha^−1^) as proposed by Plénet and Lemaire (2000):

$$\text{If }W<{\text{ 1 t ha}}^{-\text{1}},\;\%N_{\text{c}}=\text{3}.\text{4}$$ (4)

$${\text{If 1 t ha}}^{-\text{1}}\leq W\leq {\text{22 t ha}}^{-\text{1}},\;\%N_{\text{c}}=\text{ 3}.\text{4}W^{-0.\text{37}}$$ (5)

Here, the critical nitrogen concentration (%N_c_) expresses the nitrogen concentration in plant material below which total biomass (*W*) is significantly lower than the biomass that is obtained with a higher nitrogen input.

Equation (5) can also be expressed as the relationship between shoot biomass (*W*) and the (critical) nitrogen uptake (Nu_c_, kg N ha^−1^):

$$Nu_{\text{c}}=\text{ 34}W^{0.\text{63}}$$ (6)

Lemaire and Gastal (1997) proposed a nitrogen nutrition index (NNI) to quantify the intensity of both N deficiency and luxury consumption of a given crop. Nitrogen nutrition index is calculated as the ratio between the actual plant N concentration (%N_a_) of the crop and the critical nitrogen concentration (%N_c_) corresponding to the actual crop mass:

$$NNI=\frac{\%N_{a}}{\%N_{c}}$$ (7)

When NNI is close to 1, the plant N status is considered as near optimum. Departures from 1 indicate deficiency (NNI < 1) or excess nitrogen uptake (NNI > 1). In the case of deficiency, the intensity of this deficiency equals 1 − NNI. In the case of luxury N uptake, the intensity of excess equals NNI − 1 (Gastal *et al.* 2015).

A linear regression was used to analyse the relationship between leaf SPAD values and leaf nitrogen concentration (NC, mg N g^−1^ leaf), between SPAD values and SLN, and between photosynthetic rate and SLN. A hyperbolic equation (Equation (8)) was used to describe mathematically the relationship between photosynthetic rate *A* and stomatal conductance for CO_2_ (*g*_s_) and determine whether there were differences between treatments in this relationship.

$$A=A_{\text{max}}\;\times \;\frac{g_{s}}{g_{s}+g_{s50}}$$ (8)

where *A* is the observed photosynthetic rate (μmol CO_2_ m^−2^ s^−1^), *A*_max_ is the estimated maximum photosynthetic rate (μmol CO_2_ m^−2^ s^−1^), *g*_s_ is the stomatal conductance for CO_2_ diffusion (mol m^−2^ s^−1^) and *g*_s50_ is the stomatal conductance for CO_2_ at ½ *A*_max_ (mol m^−2^ s^−1^). To obtain estimates of *A*_max_ and *g*_s50_, Equation (8) was fitted to the data. To determine whether there were differences in *A*_max_ and *g*_s50_ between treatments or groups of treatments, we fitted Equation (8) to each of the five treatments separately, and also to the data of different combinations of treatments. Specifically, the data were fitted using five data sets, i.e. one data set for each treatment; using two data sets, i.e. one data set for sole maize and skip-row maize and another data set for intercrop; and one data set, i.e. one data set for the data of all treatments combined. Akaike’s Information Criterion (AIC) was used to judge the grouping of the treatment data that was best supported, with small AIC values representing better overall fits (Bolker 2008).

## Results

### Maize nitrogen and phosphorus uptake

Nitrogen uptake per unit land and per plant was significantly higher in sole maize and skip-row maize than in intercropped maize, both at flowering and at maturity (Table 2). There were no differences in nitrogen uptake between different intercrop treatments. Phosphorus uptake per unit land and per plant at flowering was significantly higher in sole maize and skip-row maize than in intercropped maize (Table 2). However, differences between treatments in phosphorus uptake had decreased at maturity, indicating recovery from initial uptake limitations in the intercrops. When measured as phosphorus uptake per unit land at maturity, sole maize had a higher phosphorus uptake than intercrops. When measured as phosphorus uptake per plant, skip-row maize had a higher phosphorus uptake than intercropped maize and sole maize. Overall, these data show that individual maize plants in intercrops had lower nitrogen and phosphorus uptake than sole maize or skip-row maize. There were no significant differences between the replacement intercrop and the add-row intercrops.

**Table 2.** Maize biomass, yield, nitrogen uptake and phosphorus uptake in different treatments during flowering and at final harvest. Biomass and yield data are from Gou *et al.* (2016), these values are for maize only; for wheat biomass and yield see Gou *et al.* (2016). Statistical comparisons (ANOVA) were made among treatments separately for each time of sampling. No shared letters denote a statistically significant difference between treatments (*P* = 0.05) using Tukey’s HSD.

Time of measurements	Treatment	Biomass		Yield		Nitrogen uptake		Phosphorus uptake	
		g m^−2^	g per plant	g m^−2^	g per plant	g N m^−2^	g N per plant	g P m^−2^	g P per plant
At flowering	SM	782 a	78.2 a	–	–	13.8 a	1.38 a	2.23 a	0.22 a
	0:2 WM	558 b	83.6 a	–	–	10.2 b	1.53 a	1.61 a	0.24 a
	6:2 WM	324 cd	48.6 b	–	–	4.20 c	0.63 b	0.66 b	0.10 b
	6:3 WM	486 bc	48.6 b	–	–	5.10 c	0.51 b	0.94 b	0.09 b
	8:2 WM	275 d	41.3 b	–	–	3.30 c	0.49 b	0.56 b	0.08 b
At maturity	SM	2142 a	251 b	1162 a	136 ab	17.4 a	1.74 a	3.94 a	0.39 ab
	0:2 WM	1734 b	300 a	934 b	161 a	16.3 a	2.44 b	3.58 ab	0.54 a
	6:2 WM	1135 d	202 c	639 cd	114 bc	8.68 b	1.30 c	2.15 c	0.32 b
	6:3 WM	1407 c	164 c	747 c	87 d	9.67 b	0.97 c	2.74 bc	0.27 b
	8:2 WM	1006 d	176 c	537 d	94 cd	7.18 b	1.08 c	2.12 c	0.31 b

### Diagnosis of nutrient limitation

The partial LER of intercropped maize was smaller than 0.67 (the relative sowing density of 6:2WM and 8:2WM; Table 1) for 6:2WM and 8:2WM treatments, and the partial LER for 6:3WM was smaller than its relative sowing density of one (Table 3). The partial LERs were higher at maturity than at flowering (Table 3). Similar results were found for partial NER and partial PER (Table 3). Thus, all these measures indicate the occurrence of recovery growth, with plants in the intercrop accumulating biomass, N and P at greater rates than sole maize plants after flowering. There was little difference in values of partial NER and partial PER. Both partial NER and partial PER were smaller than the partial LER for biomass at both flowering and maturity, indicating lower nutrient concentrations in intercropped maize than in sole maize (Table 3).

**Table 3.** Partial LER, partial NER and partial PER for maize in intercrops at flowering and at maturity. *p*LER is partial land equivalent ratio for maize biomass or yield; *p*NER is partial nitrogen uptake equivalent ratio for maize; *p*PER is partial phosphorus uptake equivalent ratio for maize, all in wheat–maize intercropping. No shared letters denote a statistically significant difference between treatments (*P* = 0.05) using Tukey’s HSD.

Time of measurements	Treatment	*p*LER biomass	*p*LER yield	*p*NER	*p*PER
At flowering	0:2 WM	0.72 a	–	0.74 a	0.72 a
	6:2 WM	0.41 bc	–	0.30 b	0.29 b
	6:3 WM	0.63 ab	–	0.37 b	0.42 b
	8:2 WM	0.35 c	–	0.24 b	0.25 b
At maturity	0:2 WM	0.81 a	0.80 a	0.94 a	0.91 a
	6:2 WM	0.53 bc	0.55 c	0.50 b	0.55 b
	6:3 WM	0.66 b	0.64 b	0.56 b	0.70 ab
	8:2 WM	0.47 c	0.46 c	0.41 b	0.54 b

The actual nitrogen concentrations (%N_a_) in maize shoots were similar to the critical nitrogen concentration in sole maize and skip-row maize, and the NNI was 1.11 for sole maize and 1.02 for skip-row maize (Table 4), indicating that the nitrogen status in these two treatments was near optimum. The actual nitrogen concentrations were, however, much lower than the critical values in all of the intercrop treatments (Table 4). The NNI values of intercropped maize ranged between 0.51 and 0.59 in the three intercrops indicating substantial nitrogen deficiency in intercropped maize (Table 4). The actual nitrogen concentration in intercropped maize was also below the value that would be critical if total biomass was corrected upward to represent a full maize crop (calculated as actual intercrop biomass divided by the relative density of 2/3 in the case of 6:2WM and 8:2WM) such that the critical nitrogen concentration would have lower value (Table 4). These measurements indicate a limitation of maize growth by nitrogen shortage. We do not have data on critical phosphorus concentrations in maize, but the similarity in NER and PER suggests that phosphorus could also be limiting in the intercrop treatments.

**Table 4.** Comparison of actual values and critical values of maize nitrogen concentration, nitrogen nutrition index and nitrogen uptake at flowering in sole and intercrops. *W* is the maize shoot biomass (t ha^−1^), %N_a_ is the actual nitrogen concentration for maize shoot, %N_c_ is the critical nitrogen concentration calculated by Equation (5), NNI is nitrogen nutrition index which is calculated with Equation (7). Nu_a_ is the actual nitrogen uptake for maize, Nu_c_ is the critical nitrogen uptake calculated with Equation (6). *W*_corr_ is the corrected maize shoot biomass for intercrops, which is calculated as the intercropped biomass divided by the relative sowing density of maize in intercropping, %N_c,corr_ is the corrected critical nitrogen concentration for intercrops calculated by *W*_corr_ and Equation (5), NNI_corr_ is the corrected nitrogen nutrition index for intercrops calculated by *W*_corr_ and Equation (7).

Treatment	*W* (t ha^−1^)	%N_a_	%N_c_	NNI	Nu_a_ (kg N ha^−1^)	Nu_c_ (kg N ha^−1^)
SM	7.82	1.76	1.59	1.11	138	124
0:2WM	5.58	1.83	1.80	1.02	102	100
6:2WM	3.24	1.30	2.20	0.59	42	71
6:3WM	4.86	1.05	1.89	0.56	51	92
8:2WM	2.75	1.20	2.34	0.51	33	64
Correction for intercrops	*W*_corr_	%N_a_	%N_c,corr_	NNI_corr_		
6:2WM	4.84	1.30	1.89	0.69		
6:3WM	4.86	1.05	1.89	0.56		
8:2WM	4.10	1.20	2.01	0.60		

### Maize nitrogen and phosphorus concentrations at organ level

At flowering, maize leaf and stem nitrogen concentrations were significantly higher in sole maize and skip-row maize than in intercropped maize, while the nitrogen concentration in the ear was only significantly different between 6:3WM on the one hand and sole maize and skip-row maize on the other hand (Table 5). Maize leaf phosphorus concentration was significantly higher in sole maize and skip-row maize than in intercropped maize. The differences in phosphorus concentration between treatments were greater in the leaf than in the stem and ear (Table 5). Maize in the 6:3WM intercrop usually had lower nitrogen and phosphorus concentrations than maize in the replacement intercrop and the 8:2WM intercrop, but the differences were mostly not significant. Concentrations of nitrogen and phosphorus in leaf and stem decreased from flowering to plant maturity and the differences between treatments in nitrogen and phosphorus concentration were in all organs smaller at maize maturity than at flowering (Table 5). The nitrogen concentration in the grain was significantly higher in the skip-row maize than in intercrops, while grain phosphorus concentration did not differ among treatments. Overall, the treatment effects on concentrations of nitrogen and phosphorus in tissues were similar in direction and relative magnitude to the treatment effects on nitrogen and phosphorus uptake per m^2^ and per plant.

**Table 5.** Maize nitrogen and phosphorus concentrations for each organ in different treatments at flowering and maturity. Statistical comparisons (ANOVA) were made among treatments for two times of sampling separately. No shared letters denote a statistically significant difference between treatments (*P* = 0.05) using Tukey’s HSD.

Treatment	Nitrogen concentration (mg N g^−1^ dry matter)				Phosphorus concentration (mg P g^−1^ dry matter)			
During flowering	Leaf	Stem	Ear		Leaf	Stem	Ear	
SM	31.1 a	11.3 a	20.0 a		3.68 a	2.21 a	3.80 a	
0:2WM	31.4 a	11.9 a	19.8 a		3.55 a	2.19 a	3.66 ab	
6:2WM	24.7 b	6.9 b	17.5 ab		2.52 b	1.47 b	3.28 bc	
6:3WM	19.2 c	6.0 b	16.8 b		2.28 b	1.57 ab	3.26 c	
8:2WM	20.7 bc	7.2 b	17.7 ab		2.28 b	1.58 ab	3.38 bc	
**At maturity**	**Leaf**	**Stem**	**Grain**	**Straw**	**Leaf**	**Stem**	**Grain**	**Straw**
SM	14.4 ab	2.5 a	11.4 b	3.7 ab	1.47 a	0.38 b	2.90 a	0.65 a
0:2WM	15.9 a	2.6 a	13.2 a	4.4 a	1.60 a	0.64 ab	3.08 a	0.98 a
6:2WM	12.8 abc	2.1 b	10.6 bc	3.7 ab	1.65 a	0.41 ab	2.81 a	0.73 a
6:3WM	10.9 c	1.9 b	9.7 c	3.3 b	1.78 a	0.90 ab	2.83 a	0.88 a
8:2WM	11.2 bc	1.8 b	10.2 bc	3.5 b	1.96 a	0.94 a	2.97 a	1.05 a

### Maize leaf traits and photosynthetic rate during flowering

Sole maize and skip-row maize had lower SLA (i.e. ‘thicker’ leaves), and higher SLN and chlorophyll content (SPAD values) than intercropped maize, but did not show higher photosynthetic rate than intercropped maize (Table 6). Except for SPAD values, the significance of treatment differences varied across the 3 weeks.

**Table 6.** Leaf traits and photosynthetic rate of maize in different treatments. NC: leaf nitrogen concentration (mg N g^−1^ leaf); *g*_s_: stomatal conductance for CO_2_ (mol m^−2^ s^−1^); *g*_w_: stomatal conductance for water (mol m^−2^ s^−1^); *A*: photosynthetic rate (μmol CO_2_ m^−2^ s^−1^). No shared letters denote a statistically significant difference (*P* = 0.05) using Tukey’s HSD. *Means the effect of Block or Week is significant, NS means the effects are not significant. *t*-tests were used to test the difference between border rows and inner row of 6:3WM treatments, for each week and for the average across the 3 weeks; differences were not significant.

Week	Treatment	SLA (m^2^ leaf g^−1^ leaf)	NC (mg N g^−1^ leaf)	SLN (g N m^−2^ leaf)	SPAD	*g*_s_ (mol m^−2^ s^−1^)	*g*_w_ (mol m^−2^ s^−1^)	*A* (μmol CO_2_ m^−2^ s^−1^)
29	SM	0.023 a	16.3 a	0.72 a	59.4 a	0.19 a	0.31 a	26.7 b
	0:2 WM	0.022 a	16.6 a	0.75 a	60.3 a	0.17 a	0.27 a	26.7 b
	6:2 WM	0.023 a	15.4 ab	0.66 ab	50.1 b	0.28 a	0.44 a	33.5 a
	6:3 WM	0.024 a	12.7 bc	0.53 bc	47.8 b	0.21 a	0.34 a	31.1 a
	8:2 WM	0.023 a	11.8 c	0.52 c	47.0 b	0.24 a	0.38 a	32.9 a
	Block	NS	NS	NS	NS	*	*	NS
30	SM	0.024 bc	16.4 a	0.68 a	59.4 a	0.11 a	0.17 a	18.8 c
	0:2 WM	0.023 c	17.0 a	0.74 a	60.9 a	0.17 a	0.27 a	22.5 bc
	6:2 WM	0.025 ab	15.4 ab	0.61 ab	50.3 b	0.21 a	0.33 a	32.6 a
	6:3 WM	0.027 a	12.1 b	0.45 c	47.0 b	0.14 a	0.23 a	24.5 bc
	8:2 WM	0.026 ab	12.1 b	0.47 bc	46.1 b	0.16 a	0.26 a	29.0 ab
	Block	^*^	NS	NS	NS	NS	NS	NS
31	SM	0.023 ab	13.8 ab	0.59 b	60.3 a	0.16 a	0.26 a	29.0 ab
	0:2 WM	0.021 c	15.0 a	0.72 a	62.9 a	0.14 a	0.23 a	26.7 b
	6:2 WM	0.022 bc	13.5 abc	0.61 ab	53.0 b	0.19 a	0.30 a	32.2 a
	6:3 WM	0.024 a	11.8 bc	0.49 b	48.0 b	0.16 a	0.26 a	30.0 ab
	8:2 WM	0.023 abc	11.5 c	0.50 b	47.5 b	0.18 ab	0.29 a	30.6 ab
	Block	NS	NS	NS	NS	NS	*	NS
Mean	SM	0.024 b	15.5 a	0.66 b	59.7 a	0.16 b	0.25 b	24.8 c
	0:2 WM	0.022 c	16.2 a	0.74 a	61.3 a	0.16 b	0.26 b	25.3 c
	6:2 WM	0.024 b	14.8 a	0.63 b	51.1 b	0.23 a	0.36 a	32.8 a
	6:3 WM	0.025 a	12.2 b	0.49 c	47.6 c	0.18 ab	0.28 ab	28.6 b
	8:2 WM	0.024 ab	11.8 b	0.50 c	46.9 c	0.19 ab	0.31 ab	30.8 ab
	Week	*	*	*	NS	*	*	*
	Block	NS	*	*	*	NS	*	NS

**Figure 2.**
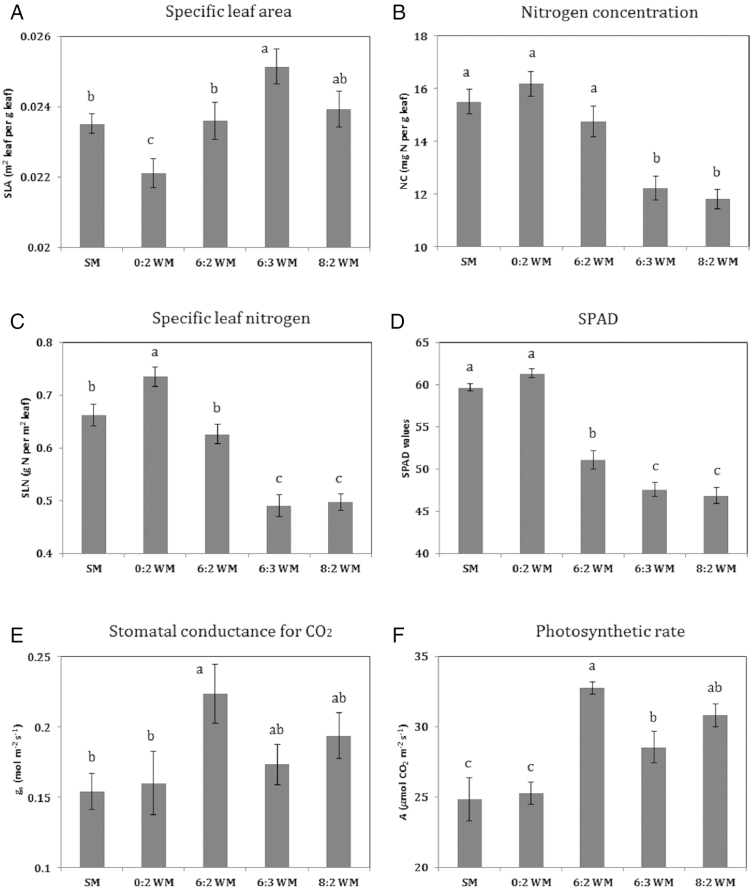
Maize ear leaf traits and photosynthetic rates in five treatments, the values are averaged over 3 weeks.

Maize grown in add-row intercrop (6:3WM) had the largest SLA (‘thinnest’ leaves) and significantly higher SLA than maize in the sole crop, skip-row or replacement intercrop, but its SLA was similar to that of the 8:2WM intercrop (Table 6). Similar patterns were observed for NC and SLN. Both parameters were significantly lower in the add-row intercrops than in the sole crop, skip-row and replacement intercrop, indicating that the stronger competition in add-row intercrops had resulted in lower N concentration. SPAD values were lower in intercrops than in sole maize or skip-row maize, when analysed separately for each week (Table 6), and maize in the add-row intercrops had lower SPAD values than maize in the replacement intercrop when averaged across the 3 weeks (Table 6; Fig. 2). The patterns in these leaf traits are consistent with the lower nitrogen uptake in intercrops as compared to the sole maize and skip-row maize. Other things being equal, these differences could result in a lowered photosynthesis rates in intercropped maize.

Stomatal conductance for CO_2_ (*g*_s_) was not significantly different between treatments in any week; however, when analysed across the 3 weeks, *g*_s_ was significantly greater in maize in the replacement intercrop than in sole maize and skip-row maize (Table 6; Fig. 2). The same was found for stomatal conductance for water (*g*_w_). The photosynthetic rate (*A*) was significantly higher in the replacement intercrop (6:2WM) than in sole maize during the first 2 weeks, but not in the third week. Overall, maize in the replacement intercrop had the highest rate of photosynthesis among all treatments, followed by maize in the add-row intercrops, and with sole maize and skip-row maize having the lowest rates of photosynthesis. All differences in parameters between border rows and inner row of 6:3WM intercrop were tested using a *t*-test, and no significant differences were found per week and for the average across the 3 weeks.

### Relationships between leaf traits and photosynthetic rate

**Figure 3.**
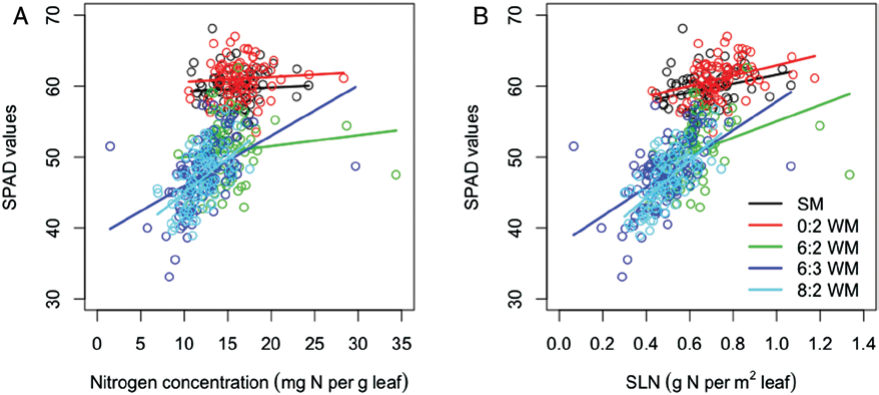
Linear regression of SPAD values on nitrogen concentration (panel A); linear regression of SPAD values on SLN (panel B).

The slopes of the linear regressions between nitrogen SPAD values and NC were not significantly different from zero for sole maize, skip-row maize and maize in replacement intercrop (Fig. 3A; **see Supporting Information—Table S2)**. The standard errors were large in all treatments. The wide range of NC and SLN in intercrops shows a strong competition for nitrogen and large variation among individual plants.

**Figure 4.**
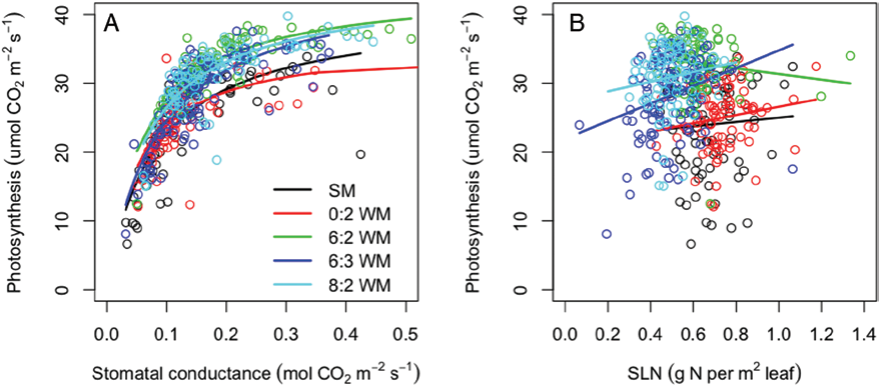
Non-linear regression (hyperbolic function) of photosynthetic rate and stomatal conductance for CO_2_ (panel A); linear regression of photosynthetic rate and SLN (panel B).

The estimated maximum photosynthetic rate (*A*_max_) from the hyperbolic function (Equation (8)) was ~43 μmol CO_2_ m^−2^ s^−1^ in intercrops, but only 39 μmol CO_2_ m^−2^ s^−1^ in sole maize and 35 μmol CO_2_ m^−2^ s^−1^ in skip-row maize **[see Supporting Information—Table S2]**. The values of stomatal conductance for CO_2_ at ½ *A*_max_ ranged from 0.051 to 0.081 μmol CO_2_ m^−2^ s^−1^ in different treatments (Fig. 4A; **see Supporting Information—Table S2)**. The curves are different from each other as the AIC was smallest when fitting the hyperbolic function separately for each individual treatment (five curves) and highest (ΔAIC = 120) when fitting the curve with data of all treatments pooled (one line). ΔAIC was 34 when fitting two curves (one curve for sole maize and skip-row maize, and another curve for intercrops). Figure 4A shows that at the same stomatal conductance, intercropped maize had a higher photosynthesis than sole maize and skip-row maize.

The slope of linear regression of photosynthetic rate (*A*) and SLN is only significant in the 6:3WM treatment (Fig. 4B; **see****Supporting Information—Table S2**). The five lines are different from each other. The AIC value was lowest for fitting five lines, and substantially higher for fitting two lines (ΔAIC = 38) or fitting only one line (ΔAIC = 127). Figure 4B shows intercropped maize has a higher rate of photosynthesis than sole maize and skip-row maize at the same SLN.

## Discussion

### The four hypotheses of this paper

The aim of this paper was to test four hypotheses: (i) maize has lower nitrogen and phosphorus uptake when grown in intercrops than in sole and skip-row maize; (ii) maize has a lower nitrogen content and chlorophyll content when grown in intercrops than as sole or skip-row maize; (iii) maize has a higher SLA when grown in intercrops than in sole and skip-row maize due to shade avoidance; (iv) maize has a lower photosynthetic rate when grown in intercrops than sole and skip-row maize due to a lower nitrogen content and a higher SLA. The first hypothesis was strongly confirmed by our data. Nitrogen uptake was significantly higher in sole maize and skip-row maize than in intercropped maize, both at flowering and at maturity; phosphorus uptake was significantly higher in sole maize and skip-row maize than in intercropped maize at flowering but not so at maturity. The second hypothesis was also confirmed by the data. Compared to sole maize, the intercropped maize had a significantly smaller chlorophyll content (SPAD values), while the NC and SLN were significantly smaller in maize in add-row intercrops, though not significantly in the replacement intercrop as compared to sole maize and skip-row maize. The findings support the notion that competition with wheat in intercrops lowers leaf nitrogen and chlorophyll content. The third hypothesis received weak confirmation. Maize SLA tended to be high in systems with strong competition (e.g. 6:3WM intercrop) and low in systems with weak competition (i.e. skip-row maize). The fourth hypothesis was, however, rejected. Intercropped maize did not have a lower photosynthetic rate (*A*) than sole maize or skip-row maize, but a higher photosynthetic rate than sole maize, especially in the replacement intercrop. Furthermore, ear leaves of maize in the replacement intercrop had a higher photosynthetic rate and stomatal conductance for CO_2_ and water than the ear leaves in sole and skip-row maize, while ear leaves in maize in the add-row intercrops had an intermediate photosynthetic rate. Overall, these findings indicate that the low biomass accumulation and low nutrient uptake in intercropped maize are partly caused by too low fertilizer application in the intercrops. No direct evidence was obtained that leaf photosynthetic rate is lowered by intercropping.

### Nutrient uptake by the crop and nutrient concentrations in organs

Smaller amounts of nitrogen and phosphorus uptake were found in intercropped maize than in sole maize, which indicates interspecific competition with wheat. Wheat is the early sown crop, and it has therefore priority in nutrient uptake. Furthermore, the yield advantage of wheat in the intercrop means that the plants take up more nutrients per plant than wheat plants in a sole crop. For example, Zhang *et al.* (2002) reported that in a wheat–maize intercrop, wheat border row plants had approximately double the nitrogen and phosphorus uptake as compared to wheat plants in a sole crop. Similarly, Zhu *et al.* (2016) found that wheat in border rows in an intercrop took up 6 g N m^−1^ row, while sole wheat took up only 2.3 g N m^−1^ row. Thus, a second crop in an intercrop with wheat experiences a reduced level of nitrogen in the soil.

During the early growth of intercropped maize, interspecific interactions lead to a decrease in biomass and nutrient acquisition (Li *et al.* 2001a), and the recovery growth after wheat harvest is crucial for yield and nutrient acquisition of intercropped maize (Li *et al.* 2001b). However, this recovery growth has to be supported by adequate nutrient supply. Li *et al.* (2010) found that yield advantage of barley–maize intercropping can only be achieved with nitrogen supply of at least 225 kg N ha^−1^. Li *et al.* (2001a) also reported that there was no recovery growth when no nitrogen and phosphorus fertilizer was applied in wheat–maize intercropping. Our diagnosis that nitrogen concentration of intercropped maize at flowering is below the critical level indicates that intercropped maize had a nitrogen deficiency that limited its biomass accumulation and yield. Thus, the low biomass accumulation and low nutrient uptake in intercropped maize are in part caused by too low fertilizer application in the intercrops. Apparently, the 200 kg N ha^−1^ of available nitrogen (168 kg from fertilizer and 32 kg ha^−1^ from residual mineral N in the soil and estimated remobilization from a cover crop) was not enough to achieve potential growth of intercrops. The given rates are in agreement with current recommendations in the Netherlands for sole spring wheat or silage maize, but appear to be limiting for the performance of a potentially high yielding intercrop. Yu *et al.* (2015) found in a global meta-analysis of LER in intercropping that LER increases with temporal complementarity between crop species (i.e. a difference in growing period resulting in competitive relaxation) and that this response to temporal complementarity is stronger at higher levels of nitrogen input. Results of the current study confirm that recommendations on plant nutrition may need to be reviewed if the potential of intercropping for high yields and high land use efficiency is to be realized.

Though nutrient acquisition by maize was significantly smaller in intercrops than in sole crops at flowering, differences in nutrient concentration at plant maturity were small, especially phosphorus concentration. Additionally, the partial LER, partial NER and partial PER were higher at maturity than at flowering. This showed that there was some recovery growth and nutrient uptake during the late growing season.

### ‘*Ex post’* hypotheses on high photosynthetic rate in intercropped maize

The strong competition with wheat resulted in leaf traits (nitrogen concentration and chlorophyll) that would in general tend to lower the capacity for photosynthesis; however, the photosynthetic rate measured in the field was not lower in the intercrops, but, on the contrary, higher. This indicates that other factors than these leaf traits and the underlying differences in nutrient concentrations in tissue affected photosynthesis. Photosynthetic rate is known to increase with SLN (Sinclair and Horie 1989; Muchow and Sinclair 1994); however, in this research this is not the case. Intercropped maize had a lower SLN but a higher photosynthetic rate than sole maize and skip-row maize. Based on the results we can propose three ‘*ex post*’ hypotheses on mechanisms that might underlie these results.

A first ‘*ex post*’ hypothesis is that intercropped maize had less water stress than sole maize or skip-row maize. This could be a consequence of the two intercropped species having peak water demand during different periods, or a changed roots distribution in the intercrop as compared to sole crops. In the wheat–maize relay intercrop, wheat is almost mature during maize flowering and the wheat plants have at that time a low water demand. There could therefore be more water available for maize in the intercrop than in sole maize. Furthermore, such niche differentiation could be amplified by below-ground root plasticity. Li *et al.* (2006) reported that the roots distributed differently in wheat–maize intercropping as compared to sole crops. Intercropped wheat spread its roots more widely than sole wheat, while intercropped maize proliferated its roots more deeply than sole maize, and both intercropped wheat and maize had a greater root length density (root length per unit soil volume) than the sole crops. Cong *et al.* (2015) reported that wheat–maize and faba bean–maize intercrops produced a greater below-ground root biomass than sole crops. Deeper and longer roots in the intercrop could support better access to ground water than is achieved in sole crops, thus the intercropped maize may experience less drought stress than sole crops. The explanation of lower drought stress in intercropped maize is supported by the observed greater stomatal conductance for water *g*_w_ in intercrops (particularly the replacement intercrops) as compared to sole crops (Table 6). Furthermore, the stomatal conductance for water in skip-row maize was also lower than that in replacement intercrop, indicating that plant plasticity in roots may play a key role in the advantage of water acquisition in the intercrop during this period. However, Fig. 4A shows that maize in intercrops had higher photosynthesis than sole crops at given stomatal conductance; hence, a difference in water status is not the full explanation for the higher than expected photosynthesis rate in intercropped maize.

**Figure 5.**
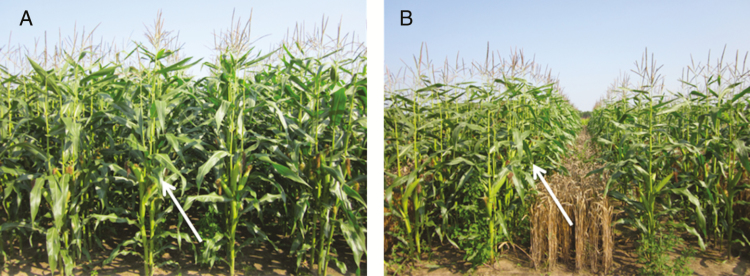
Canopy structure of sole maize (panel A) and replacement intercrop (panel B) during maize flowering stage, the arrows point the positions of the ear leaf.

A second ‘*ex post*’ hypothesis that could explain high photosynthesis rate in intercropped maize is that the ear leaves (most often the ninth leaf) of intercropped maize had a greater exposure to sunlight than the ear leaves of sole maize, due to the different canopy structure and leaf area index (LAI). Similar patterns have been found in millet–groundnut intercrop (Burgess *et al.* 2017). As a result, ear leaves in intercropped maize are likely to be acclimated to a higher light level than ear leaves in sole maize. First of all, intercropped maize allowed deeper penetration of light due to the gaps between the rows that had been planted previously with wheat. Furthermore, as shown in Fig. 5, the ear leaf is at the middle height of the plant in sole maize, and there are six to seven leaves above it. Light is strongly extinguished at this level. In the intercrop, the position of ear leaf is above the height of the wheat crop and the total amount of leaf area (in terms of LAI) above the ear leaf in the intercrop is substantially less (Fig. 5B) than in the sole crop (Fig. 5A). Due to this greater exposure to sunlight, the ear leaf of intercropped maize may have been acclimated to a higher level of radiation than the ear leaf of sole maize. Evidence contradicting this explanation is, however, the finding that the ear leaf of skip-row maize did not have a higher photosynthesis rate than the ear leaf of sole maize.

In relation to this light acclimation, nitrogen may have been allocated to different biochemical compartments within the leaf (Field 1983; Stitt and Schulze 1994). Shaded leaves contain increased levels of chlorophyll, relative to electron transport proteins and to Rubisco in order to absorb more light (Leong and Anderson 1986; Terashima and Evans 1988). We found no significant difference in SLN between replacement intercrop and sole maize, but the SPAD values were significantly lower in intercropped maize. This indicates that in the replacement intercrop, a smaller proportion of nitrogen was distributed into chlorophyll than in the sole crop, which is consistent with the higher levels of light received on the ear leaves in the intercrop as compared to the sole crop.

A third ‘*ex post*’ hypothesis is that the ear leaf may be a more important source of assimilates in intercropped maize than in sole maize. We focussed on the ear leaf because the ear leaf, due to its proximity to the ear, is likely to be an important source of assimilates for ear growth during flowering and grain filling. However, in sole maize, the ear leaf is comparatively heavily shaded, and there are six to seven leaves with better access to sunlight above it; therefore, these upper leaves may provide most of the assimilates to support plant growth. In contrast, intercropped maize has fewer and smaller leaves above the ear leaf (Zhu *et al.* 2014), and the nitrogen concentration in those leaves is lower than those in sole maize. Therefore, assimilates from these upper leaves may not be sufficient for ear growth, and the lower leaves may compensate for this.

The second and third hypotheses are in line with the higher RUE of sole maize as compared to intercropped maize. These hypotheses would also explain why the ear leaves of intercropped maize had a high photosynthesis rate, while their SLN content and chlorophyll content were lower than in the sole crops. The three hypotheses (reduced water stress, more light at ear level resulting in differences in leaf biochemistry and acclimation to light, and differences in the importance of the ear leaf in providing assimilates to the ear) could explain why the ear leaf of intercropped maize had a higher photosynthetic rate and at the same time a lower leaf nitrogen as compared to the ear leaf of sole maize. These three hypotheses were formulated ‘*ex post*’ and may be tested in new experiments.

### The relationship between leaf photosynthesis and RUE

One of the ‘*ex ante*’ hypotheses of this study was that intercropped maize would have lower photosynthesis rates than sole maize, and that this would provide an explanation for the lower RUE of intercropped maize as compared to sole maize, when considering the whole growing season (Gou *et al.* 2017). We did not find the expected association between photosynthesis rate and RUE. It should however be realized that the measurement of photosynthesis rate constituted a thin time slice of only 3 weeks around flowering, while the quantification of RUE covered the whole growing period of maize (Gou *et al.* 2017). Within this specific period of 3 weeks, photosynthesis rate was greater in intercropped maize than in sole maize, despite leaf traits in the intercropped maize (e.g. lower SLN and lower leaf chlorophyll content) that generally would support lower photosynthesis rates. Potential explanations for this discrepancy were discussed but are difficult to prove in this complex crop system. The finding of low nutrient levels in intercropped maize, below the critical level, shows that over the whole growing season, the intercropped maize did not have access to the nutrient levels that it required to attain its potential growth. Hence, one conclusion of this work is that to attain potential yield levels in wheat–maize intercropping, nutrient levels need to be raised to a level that is commensurate with crop demand, which is high due to the high radiation interception and potential production in this system (Gou *et al.* 2017). Therefore, intercrops require tailored fertilizer advice.

## Conclusions

Nitrogen and phosphorus uptake at plant level and organ level were determined at flowering and maturity in sole maize and wheat–maize intercrops, and leaf traits (SLA, NC, SLN, chlorophyll content) and photosynthetic rate of the maize ear leaf were determined in sole maize and intercrops during flowering. Intercropped maize had lower nutrient uptake and concentration than sole maize and skip-row maize, especially during flowering. We conclude that this is caused by strong interspecific competition and limiting nutrient application in intercrops. During flowering, sole maize and skip-row maize had leaf traits supporting higher photosynthetic rate than intercropped maize, e.g. smaller SLA (‘thicker’ leaves), higher SLN and chlorophyll content especially in comparison to the add-row treatments. However, the photosynthetic rate in intercropped maize was higher than in sole maize, especially in the replacement intercrop. This finding could be explained by differences between intercropped and sole maize in water availability, root traits, light distribution in the canopy, nitrogen distribution across biochemical compartments within the ear leaf and importance of the ear leaf for assimilate supply to the ear. A lower maize RUE over the whole growing season in the intercrop as found by Gou *et al.* (2017) may relate to nitrogen deficiency during grain filling. The wheat–maize intercrop system has a high potential production, and the fertilizer input needs to be tailored to the associated crop demand.

## Sources of Funding

The research described in this paper was financially supported by the China Scholarship Council (CSC), the Key Sino-Dutch Joint Research Project of NSFC (31210103906), the National Key Research and Development Program of China (2016YFD0300202) and the European Union’s Horizon 2020 Programme for Research & Innovation under grant agreement no. 727217 (ReMIX: Redesigning European cropping systems based on species mixtures).

## Contributions by the Authors

F.G., M.K.I., W.W. and L.Z. designed the field experiment and wrote the paper. F.G., A.C., Y.W., P.E.L.P. and Y.Z. conducted experiments and made measurements of photosynthesis and nitrogen content of maize leaves. All authors were involved in the discussion of experimental protocols, the interpretation of the data and approved the final submission.

## Conflict of Interest

None declared.

## Supporting Information

The following additional information is available in the online version of this article—

**Table S1**. Time schedule for leaf measurements during flowering stage.

**Table S2**. Parameters of linear or hyperbolic regressions.

Supporting Information
