# Supplementary material for: Intercropping with wheat lowers nutrient uptake and biomass accumulation of maize, but increases photosynthetic rate of the ear leaf
Source: AoB Plants. 2018 Feb 8;10(1):ply010. doi: 10.1093/aobpla/ply010 (PMC5817965; doi:10.1093/aobpla/ply010)
Supplement: Supporting Information [file ply010_suppl_supporting_information.docx]

**SUPPORTING INFORMATION**

Table S1. Time schedule for leaf measurements during flowering stage

| Week | DOY | DAE | Block | Number of plants per row | Adaptation time (min) |
| --- | --- | --- | --- | --- | --- |
| 29 | 197 | 58 | 1, 2 | 4 | 5 |
| 29 | 198 | 59 | 3, 4 | 4 | 5 |
| 30 | 204 | 65 | 1, 2 | 4 | 5 |
| 30 | 205 | 66 | 3, 4 | 4 | 5 |
| 31 | 211 | 72 | 1,2 | 2 | 15 |
| 31 | 212 | 73 | 3 | 2 | 15 |
| 31 | 213 | 74 | 4 | 2 | 15 |

Week: week of the year, DOY: day of year, DAE: day after maize emergence.

Table S2. Parameters of linear or hyperbolic regressions

| Treatment | SPAD ~ NC | SPAD ~ SLN | *A* ~ *g_s_* | | *A* ~ SLN |
| --- | --- | --- | --- | --- | --- |
|  | S1 | S2 | *A*_max_  (*μ*mol CO_2_ m^-2^ s^-1^). | *g_s50_*  (mol m^-2^ s^-1^). | S3 |
| SM | 0.056 ± 0.12 | 6.23 ± 2.36^*^ | 39.31 ± 2.01^*^ | 0.080 ± 0.011^*^ | 3.15 ± 6.11 |
| 0:2 WM | 0.068 ± 0.11 | 7.34 ± 2.23^*^ | 35.35 ± 1.67^*^ | 0.051 ± 0.008^*^ | 5.99 ± 4.22 |
| 6:2 WM | 0.015 ± 0.15 | 11.49 ± 3.42^*^ | 42.73 ± 1.00^*^ | 0.058 ± 0.006^*^ | -4.08 ± 3.51 |
| 6:3 WM | 0.71 ± 0.13^*^ | 20.19 ± 2.77^*^ | 43.61 ± 1.48^*^ | 0.081 ± 0.008^*^ | 12.83 ±4.21^*^ |
| 8:2 WM | 0.98 ± 0.17^*^ | 26.33 ± 3.29^*^ | 42.70 ± 1.50^*^ | 0.064 ± 0.008^*^ | 7.06 ± 5.62 |

S1, S2 and S3 are the slopes of linear regression of SPAD values and nitrogen concentration (NC), SPAD values and SLN, and photosynthetic rate (*A*) and SLN, respectively; *A_max_* is the maximum photosynthetic rate estimated by the hyperbolic function (*Eq*. 1), and *g_s50_* is the stomatal conductance for CO_2_ at ½ *A_max_.*^*^Asterisks represent the estimated values are significantly different from 0 (P = 0.05).
